# X-CHIP: an integrated platform for high-throughput protein crystallization and on-the-chip X-ray diffraction data collection

**DOI:** 10.1107/S0907444911011589

**Published:** 2011-05-12

**Authors:** Gera Kisselman, Wei Qiu, Vladimir Romanov, Christine M. Thompson, Robert Lam, Kevin P. Battaile, Emil F. Pai, Nickolay Y. Chirgadze

**Affiliations:** a Campbell Family Cancer Research Institute, Ontario Cancer Institute, Princess Margaret Hospital, University Health Network, Toronto, Ontario M5G 2C4, Canada; b Hauptman–Woodward Medical Research Institute, IMCA-CAT, Advanced Photon Source, Argonne National Laboratory, Argonne, Illinois 60439, USA; c Departments of Biochemistry, Molecular Genetics and Medical Biophysics, University of Toronto, Toronto, Ontario M5S 1A8, Canada; d Department of Pharmacology and Toxicology, University of Toronto, Toronto, Ontario M5S 1A8, Canada

**Keywords:** protein crystallization devices, *in situ* X-ray analysis, crystallization, crystal visual inspection, diffraction data collection

## Abstract

The X-CHIP (X-ray Crystallization High-throughput Integrated Platform) is a novel microchip that has been developed to combine multiple steps of the crystallographic pipeline from crystallization to diffraction data collection on a single device to streamline the entire process. The system has been designed for crystallization condition screening, visual crystal inspection, initial X-ray screening and data collection in a high-throughput fashion. X-ray diffraction data acquisition can be performed directly on-the-chip at room temperature using an *in situ* approach. The capabilities of the chip eliminate the necessity for manual crystal handling and cryoprotection of crystal samples, while allowing data collection from multiple crystals in the same drop. This technology would be especially beneficial for projects with large volumes of data, such as protein-complex studies and fragment-based screening. The platform employs hydrophilic and hydrophobic concentric ring surfaces on a miniature plate transparent to visible light and X-rays to create a well defined and stable microbatch crystallization environment. The results of crystallization and data-collection experiments demonstrate that high-quality well diffracting crystals can be grown and high-resolution diffraction data sets can be collected using this technology. Furthermore, the quality of a single-wavelength anomalous dispersion data set collected with the X-CHIP at room temperature was sufficient to generate interpretable electron-density maps. This technology is highly resource-efficient owing to the use of nanolitre-scale drop volumes. It does not require any modification for most in-house and synchrotron beamline systems and offers a promising opportunity for full automation of the X-ray structure-determination process.

## Abstract 2

The X-CHIP (X-ray Crystallography High-throughput Integrated Platform) is a novel microchip that has been developed to combine multiple steps of the crystallographic pipeline from crystallization to diffraction data collection on a single device to streamline the entire process.

## 1. Introduction

High-throughput protein crystallography can be a time-consuming and resource-intensive endeavor. Although recent years have seen many advances in the field, screening for suitable crystallization conditions using common commercially available platforms still requires considerable amounts of protein and reagents. Furthermore, diffraction-quality testing and data collection typically involve physical extraction and cryogenic freezing of the crystal samples, which may have a significant impact on the integrity of the crystal (Garman, 1999 ▶). To acquire high-quality diffraction data, both the crystallization conditions and the cryoprotectants must be further optimized. These steps can be time-consuming and are often restricted to experienced users (Alcorn & Juers, 2010 ▶). In response to these concerns, the last decade has seen a significant surge of developments in crystallography-aimed microtechnology, specifically the use of crystallization chips. To date, the field has been dominated by a range of microfluidic devices (Li & Ismagilov, 2010 ▶), with one of the most significant differences between them being the type of crystallization technique that they employ. Several devices have been developed and even commercialized (such as the Topaz crystallizer from Fluidigm Corp., California, USA and The Crystal Former from Microlytic Inc., Massachusetts, USA) that utilize free-interface diffusion (FID; Hansen *et al.*, 2002 ▶). Other chips employ nanochannels to create counter-diffusion crystallization (Hasegawa *et al.*, 2007 ▶; Ng *et al.*, 2008 ▶; Dhouib *et al.*, 2009 ▶) or nanodroplets that simulate batch crystallization (Zheng *et al.*, 2003 ▶). There are two clear parallel implications in all these devices. They are all striving to increase the efficiency of the hit-identification process and are offering the possibility of *in situ* X-ray analysis and, in favorable cases, diffraction data collection for structure determination (Zheng *et al.*, 2004 ▶; Hansen *et al.*, 2006 ▶; Ng *et al.*, 2008 ▶; May *et al.*, 2008 ▶; Dhouib *et al.*, 2009 ▶)

The X-CHIP^1^ (Chirgadze *et al.*, 2009 ▶) addresses the same challenges of high-throughput crystallography using an alternative approach and has a number of unique additional advantages. In contrast to microfluidic chips, the crystallization process takes place on the chip surface in droplet arrays of aqueous protein and crystallization reagent mixtures under a layer of oil. These microbatch arrays are made possible by altering the chip surface with a unique coating, creating defined areas of varying hydrophobicity. This paper presents the design of the device and accompanying tools for setting up crystallization trials and mounting the chip for data collection, as well as the important benefits, limitations and implications that are inherent to this platform. It also describes proof-of-concept experiments in which this technology was utilized for crystal growth, visual inspection, X-ray diffraction data collection and structure determination of two native and one selenomethionine-labeled protein targets. The presented results show that large well diffracting crystals can be grown and high-quality data sets sufficient for structure determination can be collected on a home as well as a synchrotron X-ray source.

## 2. X-CHIP design and application

**Figure 1:**
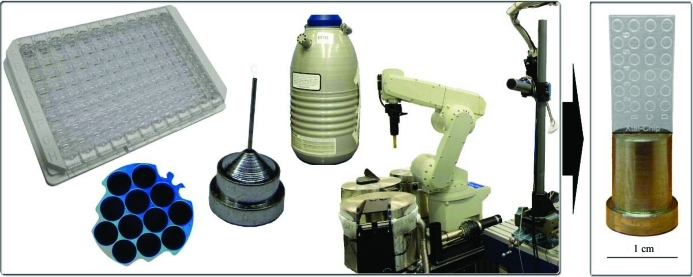
The X-CHIP was designed and developed as an alternative to the conventional stages of the existing crystallization to data collection process.

**Figure 2:**
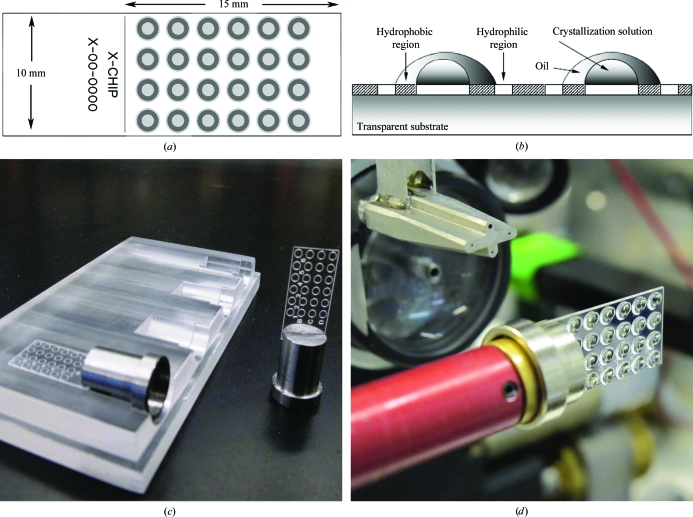
Schematics and images of the X-CHIP. (*a*) Top-view schematic of the 24-drop format chip; hydrophilic and hydrophobic areas are shown in light and dark gray, respectively (other formats include six and 48 drops; not shown). (*b*) Cross-section of the chip. (*c*) X-CHIP on a base and a four-chip receptacle device. (*d*) X-CHIP with 24 crystallization drops mounted on a goniometer.

The principal idea behind the X-CHIP was to create a platform that presents an alternative to the conventional crystallo­graphic pipeline by placing crystallization condition screening, crystal inspection and data collection onto one device, streamlining the entire process and eliminating crystal handling and arduous cryogenic techniques (Fig. 1 ▶). The chip is made from a material chosen for its visual light transparency and relatively low absorption of X-ray radiation. An X-CHIP with a thickness of 0.375 mm absorbed approximately 30% of the X-ray intensity of the primary synchrotron beam, which was attenuated 1800–2000 times to avoid excessive radiation damage to the crystal during data acquisition. Designed to be compatible with most standard goniometers, the device inserts into a chip-base (possessing a machined slot) for support and simple mounting. A plastic receptacle holds multiple chips mounted on bases, providing rigidity for setup, storage and visual inspection of the crystallization drops, and can be covered with a special lid to prevent dust contamination. The chip, along with supporting tools, is shown in Fig. 2 ▶.

**Figure 3:**
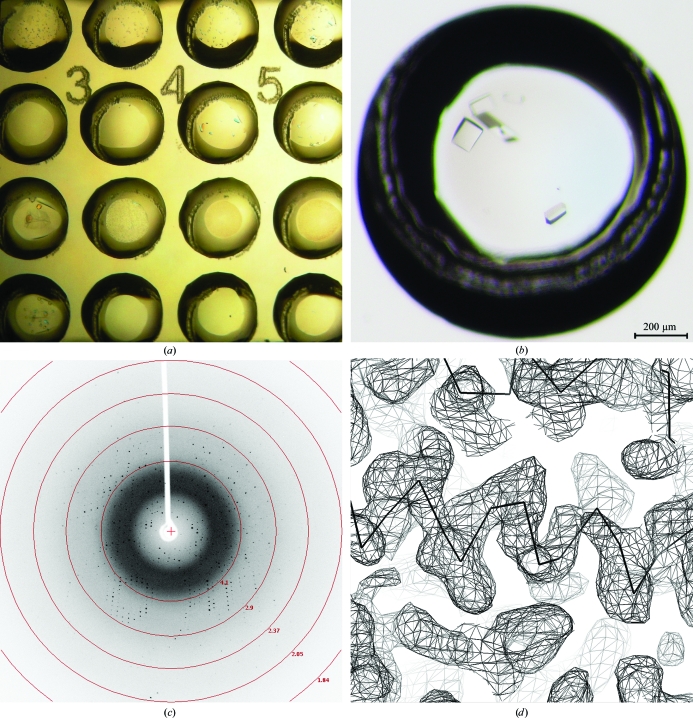
Experimental results. (*a*) Section of a 4 × 6 X-CHIP with a two-dimensional optimization of two crystallization conditions for native PA0269 taken two weeks after initial setup. (*b*) Crystals of EphA3 grown overnight and the crystals were approximately 150 µm in length. (*c*) On-the-chip diffraction image for an EphA3 crystal collected on a Rigaku FR-E rotating-anode generator using an R-AXIS HTC detector. (*d*) Part of an experimental electron-density map generated using the SAD PA0269SM data set collected directly from the crystal grown on the X-CHIP superimposed with the protein Cα trace (shown as a solid line).

The system described applies principles of the microbatch crystallization method, the high effectiveness and unique benefits of which have been described elsewhere (D’Arcy *et al.*, 1996 ▶, 2003 ▶; Chayen, 1998 ▶). On the surface of the chip, circular hydrophilic areas are inscribed in hydrophobic annuli in ordered arrays (Figs. 2 ▶ *a* and 2 ▶ *b*). Nanolitre volumes of aqueous protein and precipitant solutions are mixed onto the hydrophilic circle by sequential addition and then covered by an oil layer, which is dispensed on top of the drop and is stabilized on the surrounding hydrophobic ring. The inter­actions between the aqueous phase, oil layer and coated surface create highly defined droplets of predictable volume and thickness and prevent drops from fusing with each other during crystallization setup and data acquisition. The design of the chip currently uses 1 × 6, 4 × 6 or 4 × 12 formats and its size permits visual inspection of the entire chip in one image (Fig. 3 ▶ *a*).

## 3. Materials and methods

Previously investigated targets, the protein kinase domain of human ephrin receptor tyrosine kinase A3 (EphA3; Davis *et al.*, 2008 ▶) and the *Pseudomonas aeruginosa* alkylhydroperoxidase D protein (PA0269; PDB entry 2o4d; Clarke *et al.*, 2011 ▶), were selected as model proteins to demonstrate the feasibility of crystal growth and *in situ* data collection using the X-CHIP. Following several rounds of on-chip optimization, both projects were crystallized under conditions similar to those in the cited literature. EphA3 at 15 mg ml^−1^ and native PA0269 at 10 mg ml^−1^ were crystallized at a 1:1 ratio with 0.2 *M* ammonium sulfate, 0.1 *M* HEPES pH 7.5, 25% PEG 3350 and 0.8 *M* ammonium sulfate, 0.1 *M* sodium citrate pH 4.0, respectively. For crystallization-drop setup, a protein-sample volume of 200–250 nl was mixed with an equal volume of precipitant solution and then covered by an oil volume of 0.75–1.25 µl; all solutions were dispensed with a Gilson P2 pipette. Prior to setup, the chip was inserted into the holder, which was subsequently covered with a lid to prevent contamination. Chips were stored for 1–4 weeks without significant evaporation and were transported by road to the synchrotron beamline. Crystals from both stored chips as well as chips set up at the synchrotron were used to collect diffraction data sets.

In-house data sets were collected on a Rigaku FR-E Superbright rotating-anode X-ray source equipped with a Rigaku R-AXIS HTC image plate as a detector (Rigaku, The Woodlands, Texas, USA). The synchrotron data sets were collected on the IMCA-CAT ID-17 beamline at the Advanced Photon Source (APS) facility with appropriate beam attenuation using a Pilatus 6M detector (Dectris Ltd, Baden, Switzerland). The X-CHIP was manually mounted onto the goniometers as shown in Fig. 2 ▶(*d*). Individual samples were initially optically centered and then centered using diffraction to refine the crystal position. In both cases the cryostream was blocked and data collection was performed at room temperature.

## 4. Results

Two important aspects of the described system were investigated throughout this study; the capacity of the chip to pro­duce good-quality crystals and the feasibility of the acquisition (*in situ*) of diffraction data of sufficient quality for *de novo* structure determination. To assess the first task, the reproducibility of previous hits obtained by the sitting-drop vapor-diffusion technique was tested. For both the EphA3 and PA0269 projects, vapor-diffusion crystallization conditions resulted in high-quality crystals on the X-CHIP (Figs. 3 ▶ *a* and 3 ▶ *b*). For PA0269, on-chip optimization further improved the crystal size and quality and decreased the number of crystals per drop (Fig. 3 ▶ *a*). These results demonstrate that the X-CHIP can be successfully used to obtain and optimize crystallization hits and grow single crystals that are large enough for straightforward data collection.

Proof-of-concept experiments for on-the-chip data collection were carried out on the rotating-anode source and the synchrotron beamline. Initial data-collection trials using the in-house X-ray source led to the acquisition of a complete EphA3 data set. While the experiment was conducted at room temperature, diffraction data could still be obtained with sufficient completeness, even for crystals of such a low-symmetry space group as *P*2_1_ (Table 1 ▶). On the synchrotron beamline, data sets were collected for EphA3, PA0269 and a PA0269 selenomethionine derivative (SAD). The high sensitivity and ultrafast readout time of the Pilatus 6M detector allowed complete data sets to be collected quickly at room temperature without significant degradation of the sample and with excellent processing statistics. Owing to the finely focused beam (50 × 50 µm), it was possible to collect data from multiple small crystals grown within the same drop without any obvious impact on the diffraction quality of neighboring crystals. A particularly interesting result can be observed by comparing the mosaic spread between the X-CHIP and the benchmark data (*i.e.* CryoLoop) in Table 1 ▶. It is evident that the mosaic spread was consistently lower for data sets collected using the chip and in the case of PA0269 was as low as 0.046°. Furthermore, based on resolution range alone, EphA3 crystals only started showing radiation damage after as long as 10 min of continuous X-ray exposure, which was more then twice the time needed to obtain a full data set (data not shown).

For crystallization of EphA3 and PA0269, paraffin oil was used to coat the crystallization drops after protein and precipitant solutions had been dispensed. Other oils have been explored, such as Al’s Oil (Hampton Research; a 50/50 mixture of paraffin oil and silicone oil), silicone oil and a 50/50 mixture of Paratone and paraffin oils. Higher viscosity oils (paraffin and Paratone/paraffin) performed better on the X-­CHIP by being highly restricted to the hydrophobic ring boundaries. The thinner silicone oil was found to flow outside of these boundaries, causing drops to merge. Al’s Oil required more careful application compared with higher viscosity oils, but proved to stay within the hydrophobic boundaries. Crystallization conditions containing ethanol, 2-methyl-2,4-pentane­diol (MPD) and detergents were also tested on the X-CHIP. Ethanol tolerance was tested with a 5–30% gradient using paraffin oil as a cover. The phase separation within the crystallization drops remained intact over the entire gradient range. Crystallization drops containing MPD in combination with different oils tolerated up to 8% MPD before they began to spill over into the hydrophobic area. While this may exclude some MPD-based conditions from being used on the X-CHIP, the impact on the overall versatility is low since for most initial screens available from Hampton Research and Emerald BioSystems (*e.g.* Wizard I and II, Index and Crystal Screen) only about 5% of the total conditions contain MPD. Detergent tolerance was tested with *n*-dodecyl-*N*,*N*-dimethyl­amine-*N*-oxide (DDAO) and *n*-octyl-β-d-glucoside in combination with a paraffin-oil covering. Separation between the phases remained intact with 0.05% *n*-octyl-β-d-glucoside but DDAO was not tolerated even at very low concentrations.

## 5. Discussion

The series of initial experiments on the X-CHIP crystallization platform described above demonstrates the chip’s applicability for high-throughput protein crystallography and provides insight into the benefits and limitations of this system. Crystallization using the microbatch method on the chip was shown to be suitable for crystal growth and also offered additional benefits. Oil-covered drops evaporate very slowly (days to weeks), simplifying both manual and automated setup. Furthermore, changing the composition of the top oil layer with various oil mixtures makes it possible to vary the rate of water evaporation over a wide range, adding another favorable dimension to crystallization screening (D’Arcy *et al.*, 2004 ▶). Inherently, the system is economical since crystallization-hit determination and optimization trials require up to five times less protein sample by volume and 500 times less reagent solution than standard vapor-diffusion methods. Theoretically, the volumes can be decreased even further by applying robotic liquid-handling systems, but are currently limited by the accuracy of manual dispensing. In addition, the simplicity of the device results in low manufacturing costs and the platform design eliminates the time and expense associated with cryogenic techniques. The small size of the chip offers more convenient and faster visual inspection, as all of the crystallization drops can be viewed simultaneously under a microscope. Furthermore, the system design provides a non-invasive means of diffraction testing and screening, as the developed device can be mounted on most in-house and synchrotron-beamline data-acquisition systems without any modification of the chip or adjustments to the system. These capabilities of the X-CHIP make it a potentially useful platform for high-throughput initiatives such as fragment-based screening by co-crystallization.

The X-CHIP system has the potential to completely remove the ‘user factor’ between crystal growth and X-ray diffraction data collection, eliminating crystal manipulation. The feasibility of *in situ* data collection has significant implications. Firstly, data collection at room temperature eliminates the need for the tedious and often limiting step of cryocondition optimization and results in crystal structures that are determined at temperatures that are more relevant to the physiological state. Additionally, experimentally obtained SAD data display excellent processing statistics that are clearly of sufficient quality for *de novo* structure determination. Interestingly, in at least one of the cases investigated, undisturbed crystals showed a significantly lower mosaic spread than those of cryogenically frozen samples, suggesting the potential application of this system to samples of high sensitivity or those with a large unit cell (Table 1 ▶). Once mounted on the goniometer, navigation along the chip and alignment of any crystal in the drops is quite straightforward, presenting the potential for data collection in a high-throughput mode. This approach eliminates the down-time necessary for mounting of individual loops as in conventional robotic systems and may save hours of valuable synchrotron beam time. Finally, the elimination of manual crystal handling opens up the opportunity for full automation of the crystallization to data acquisition pipeline to streamline the entire process.

Current developments on the project are aimed at scaling down the drop volumes of the X-CHIP system. Attempting to do so using a manual setup has proven to be challenging, but application of a liquid-handling robotic system may address this issue. The Mosquito crystallization robotic system (Molecular Dimensions Ltd, Suffolk, England) has already been used to successfully set up crystallization experiments with total drop volumes as low as 200 nl. The X-CHIP is also being applied to the crystallization trials of additional protein targets. As a point of interest, experiments with highly sensitive and/or small crystal samples could greatly benefit from the use of this system, as the non-invasive data-collection approach would be likely to resolve many problems that arise from crystal handling. We are also exploring the application of the chip in projects in which low mosaic spread is essential for a successful outcome.

## 6. Conclusions

From the initial studies of the device it is evident that not only does the X-CHIP have the potential to increase efficiency and offer on-the-chip *in situ* data collection for *de novo* structure determination, but also has a range of additional benefits including the opportunity for full automation. Even though the recent growth of microchip crystallization technology has seen the development of several useful devices, the X-CHIP platform offers previously unprecedented simplicity with comparable or even better performance. Its intuitive design, minimalistic support platform and compatibility with most beamlines make this device an attractive tool for protein crystallization and X-ray diffraction data collection.

## Figures and Tables

**Table 1:** Summary of selected data sets Values in parentheses are for the highest resolution shell. Benchmark data are shown in italics.

	EphA3	*EphA3*	EphA3	*PA0269SM*†	PA0269	PA0269SM†
Crystal is in/on	X-CHIP	*CryoLoop*	X-CHIP	*CryoLoop*	X-CHIP	X-CHIP
Sample temperature (K)	295	*100*	295	*100*	295	295
X-ray source	Rigaku FR-E	*BM-17 APS*	ID-17 APS	*ID-17 APS*	ID-17 APS	ID-17 APS
Wavelength (Å)	1.54	*1.00*	1.00	*0.97934*	1.00	0.97938
Detector	R-AXIS HTC	*MAR 300 CCD*	Pilatus 6M	*ADSC Q210r*	Pilatus 6M	Pilatus 6M
Space group	*P*2_1_	**P*2_1_*	*P*2_1_	**P*6_3_22*	*P*6_3_22	*P*6_3_22
Resolution (Å)	2.00 (2.10–2.00)	*1.93 (2.03–1.93)*	1.95 (2.05–1.95)	*1.75 (1.84–1.75)*	1.95 (2.05–1.95)	1.95 (2.05–1.95)
Data-collection time (min)	100	*29*	5.3	*74*	3.0	3.3
Δϕ_total_ (°)	100‡	*190*	160	*185*	90	100
Mosaic spread (°)	0.100	*1.048*	0.360	*0.364*	0.046	0.160
Completeness (%)	85.3‡	*98.4*	96.2	*99.8*	100	100
Multiplicity	2.4	*3.6*	2.7	*10.6*	9.5	10.3
〈*I*/σ(*I*)〉	4.5 (2.2)	*12.9 (5.0)*	7.5 (2.7)	*19.4 (3.5)*	12.1 (2.7)	20.8 (3.9)
*R*_merge_ (%)	8.8 (34.6)	*4.9 (9.7)*	10.9 (34.6)	*7.5 (49.9)*	8.2 (47.5)	6.9 (50.2)

† Single-wavelength anomalous dispersion (SAD) data collection using anomalous signal from selenomethionine.

‡ A completeness of 99% was achievable from the same crystal with a total oscillation angle of 140°.

## Footnotes

1 For any inquires about the X-CHIP please contact the corresponding author.
